# IgG4-related disease as an uncommon cause of myocarditis: a case report

**DOI:** 10.1093/ehjcr/ytag274

**Published:** 2026-04-30

**Authors:** Emily Ellis, Sarah Grebennikov, Rodolfo Jose Denadai Benatti

**Affiliations:** Internal Medicine, Riverside Methodist Hospital, 3535 Olentangy River Road, Columbus, OH 43214, USA; Internal Medicine, Riverside Methodist Hospital, 3535 Olentangy River Road, Columbus, OH 43214, USA; Advanced Heart Failure and Transplant Cardiology, Riverside Methodist Hospital, 3535 Olentangy River Road, Columbus, OH 43214, USA

**Keywords:** IgG4-related disease (IgG4-RD), Myocarditis, Myocardial injury, Non-ischaemic fibrosis, Autoimmune disease, Case report

## Abstract

**Background:**

IgG-4-related disease (IgG4-RD) is a systemic fibroinflammatory disease that can affect any organ system. Myocarditis is a rare manifestation.

**Case summary:**

A 65-year-old woman with clinically diagnosed IgG4-RD, who had recently completed corticosteroid taper, presented with multiple admissions for recurrent chest pain and myocardial injury. Coronary angiography repeatedly showed preserved coronary anatomy, and initial transthoracic echocardiography showed preserved left ventricular systolic function. Subsequent transthoracic echocardiography showed rapid decline in ejection fraction, and cardiac magnetic resonance imaging (MRI) showed myocardial oedema, infarct scar, and patchy non-ischaemic fibrosis. A probable diagnosis of IgG4-RD myocarditis was made clinically based on medical history, multimodal imaging findings, and exclusion of alternative diagnoses, although tissue confirmation was not obtained. The patient improved clinically and radiographically with corticosteroid re-initiation followed by maintenance with mycophenolate mofetil (MMF).

**Conclusion:**

This case underscores the importance of considering IgG4-RD in the differential diagnosis of unexplained myocardial inflammation and highlights the potential reversibility of cardiac injury with early immunosuppression.

Learning points

- IgG4-related disease should be considered in patients with troponin-positive non-obstructive coronary arteries and a history of systemic inflammatory disease.
- Cardiac MRI can distinguish ischaemic from non-ischaemic injury patterns; however, alternative diagnoses should be excluded before attributing findings to IgG4-RD.

## Introduction

IgG4-related disease (IgG4-RD) is an immune-mediated condition characterized by infiltration of IgG4-positive plasma cells, storiform fibrosis, and obliterative phlebitis affecting multiple organ systems, most commonly salivary glands, thyroid, lungs, pancreas, kidney, retroperitoneum, and biliary tract.^1^ Cardiovascular involvement is uncommon, with myocardial infiltration causing myocarditis being particularly rare.^2-4^

## Summary figure

Clinical timeline of IgG4-related disease-associated myocarditis

**Table:** 

Time/interval	Clinical events	Diagnostic findings	Treatment/outcome
Five months prior	Submandibular swelling and globus sensation	WBC 15.00 K/µl with 37% eosinophils; serum IgG4 132 mg/dl (ref: 4–86); unrevealing haematologic and cross-sectional imaging workup	Prednisone with prolonged taper initiated for probable IgG4-related disease, with symptom resolution and normalization of eosinophilia
Two months after steroid taper, Day 0	Recurrent chest pain, initial admission	Troponin 213–257 ng/L; patent prior stents on coronary angiography; LVEF 55%	Discharged on indefinite clopidogrel therapy
Day 3	Recurrent chest pain, readmission	Troponin 1277 ng/L; repeat angiography with patent stents; preserved LVEF	Continued inpatient observation
Day 5	Persistent angina during the same admission	Troponin peak 2490 ng/L	Repeat LHC with placement of two additional DES proximal to prior stents
Day 7	Recurrent chest pain, readmission	Troponin 2102 ng/L; NT-proBNP 5046 pg/ml; LVEF 35% with new anterior/lateral hypokinesis; cardiac MRI with myocardial oedema, inflammation, patchy non-ischaemic fibrosis; IgG4 87.9 mg/dl (normal)	Rheumatology consulted; prednisone 30 mg daily restarted with taper; GDMT initiated
Three-month follow-up	Clinical improvement	Repeat cardiac MRI with resolution of myocardial oedema and inflammation	Mycophenolate mofetil maintenance

Diagnosis of IgG4-RD can be challenging due to clinical overlap with oncologic and autoimmune disorders. The gold standard is tissue biopsy confirming IgG4-positive plasma cell infiltration with characteristic histopathological features. When biopsy is not feasible, diagnosis relies on clinical features, serology, imaging, and exclusion of alternative diagnoses according to the 2019 ACR/EULAR classification criteria.^5^ However, serum IgG4 concentrations may be normal in approximately 30% of patients with histopathologically confirmed disease and can be elevated in various non-IgG4-RD conditions, limiting their diagnostic utility as a standalone marker.^6,7^ We present a case of clinically suspected IgG4-related myocarditis in a patient with recurrent myocardial injury and preserved coronary anatomy, with marked improvement following corticosteroid therapy.

## Case presentation

A 65-year-old female with past medical history of coronary artery disease with drug-eluting stents to the left anterior descending coronary artery and circumflex arteries, hypertension, and paroxysmal atrial fibrillation presented with 5 weeks of submandibular swelling and globus sensation. Otolaryngologist evaluation revealed pachydermia with reflex and postnasal drip, and symptomatic care was recommended.

Evaluation also incidentally revealed leukocytosis (WBC 15.00 K/µl) with 37% eosinophils (absolute eosinophil count of 5.82 k/µl), haemoglobin 12.7 g/d, and platelet count 383 K/µl. Peripheral smear showed increased mature eosinophils including trilobed forms. She was referred to haematology for further evaluation and a comprehensive workup including Janus kinase 2 (JAK2) mutation analysis, and imaging with computed tomography of chest, abdomen, and pelvis was unremarkable.

The constellation of submandibular gland swelling, peripheral eosinophilia, and the absence of clonal haematologic findings raised suspicion for an immune-mediated process. Workup revealed an elevated serum IgG4 concentration of 132 mg/dl (reference range: 4–86 mg/dl).

The clinical picture appeared consistent with probable IgG4-RD despite lack of tissue confirmation, and she was started on prednisone 40 mg daily for 2 weeks followed by a prolonged taper, resulting in complete symptom resolution and normalization of eosinophilia.

Several months after completing the steroid taper, she presented with recurrent chest pain and troponin elevation from 213 to 257 ng/L. Coronary angiography showed widely patent stents without new obstructive lesions. The echocardiogram revealed preserved left ventricular ejection fraction (55%). She was discharged on indefinite clopidogrel therapy after symptomatic improvement.

Three days later, she was readmitted with recurrent chest pain and troponin elevated to 1277 ng/L. Repeat coronary angiography again demonstrated widely patent stents, and repeat echocardiogram was unchanged.

During the same hospitalization, given persistent angina along with rising troponins peaking at 2490 ng/L, she underwent a third left heart catheterization. This resulted in the placement of two 3.0 mm drug-eluting stents just proximal to her previously placed stents. She was discharged in stable condition.

**Figure 1:**
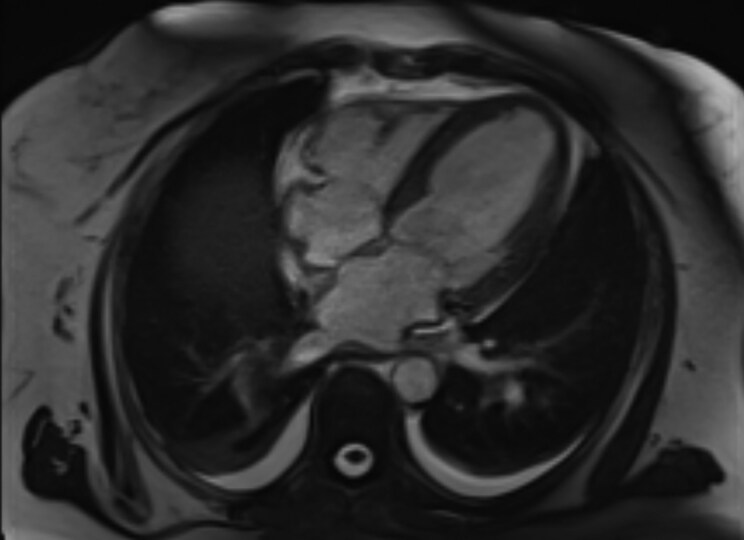
Four-chamber CINE cardiac MRI demonstrating myocardial oedema and inflammation in the acute phase. Findings include increased T2 signal in affected segments consistent with acute myocardial oedema.

The following day, she presented with recurrent chest pain. Troponin was elevated to 2102 ng/L with concomitant elevation of NT-Pro BNP to 5047 pg/ml. Repeat echocardiogram on admission showed reduced ejection fraction of 35% and new severely hypokinetic anterior/lateral walls. Cardiac MRI showed evidence of myocardial oedema and inflammation along with inferior/inferolateral wall infarct scar with patchy non-ischaemic fibrosis. Findings were suggestive of infarct, inflammatory myopathy, and stress-induced cardiomyopathy (*Figure 1*).

**Figure 2:**
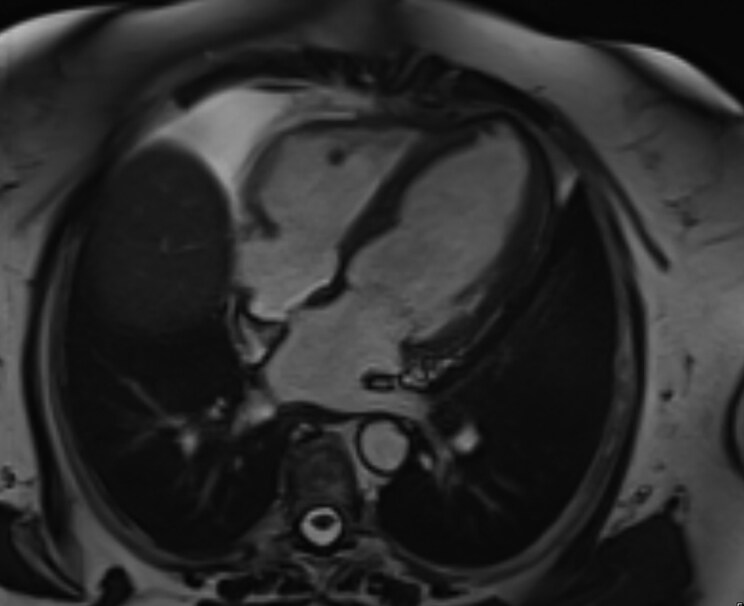
Four-chamber CINE cardiac MRI at 3-month follow-up demonstrating continued but improved myocardial inflammation and oedema compared to prior imaging, with no new non-ischaemic fibrosis.

Given the history of probable IgG4-RD with case reports of IgG4-RD being implicated in causing myocarditis, rheumatology evaluated the patient during hospitalization. Repeat labs included an IgG4 level of 87.9 (reference range: 2.4–121.0 mg/dl). Given high enough clinical suspicion, she was discharged on prednisone 30 mg daily with a planned taper over 2–3 weeks, leading to overall symptomatic improvement. Guideline-directed medical therapy (GDMT) was started, and she was referred to the structural heart team outpatient for continued management. During haematology follow-up, immunosuppressive therapy with MMF 1000 mg twice daily was started for maintenance treatment. Repeat cardiac MRI at 3 months demonstrated improvement of myocardial oedema and inflammation without new evidence of non-ischaemic fibrosis, indicating a favourable response to therapy (*Figure 2*).

## Discussion

Patients presenting with elevated troponins and myocardial injury without obstructive lesions found on left heart catheterization are labelled as having troponin-positive non-obstructive coronary arteries (TpNOCA). The differential diagnosis is broad, including ischaemic (coronary plaque disruption, coronary artery dissection, coronary vasospasm, and microvascular dysfunction) and non-ischaemic causes (myocarditis, stress-induced cardiomyopathy, other cardiomyopathies, and extracardiac conditions).^8^ Post-percutaneous coronary intervention (PCI) myocardial injury should be considered in patients with recent stent placement. Inflammatory conditions such as IgG4-RD are rarely considered early in workup, leading to delays in targeted therapy.

In this case, alternative diagnoses were systematically considered. Stress-induced cardiomyopathy was a reasonable consideration given the apical ballooning pattern; however, the recurrent nature of symptoms, temporal relationship to corticosteroid withdrawal, and response to immunosuppression favoured an inflammatory aetiology. Post-PCI myocardial injury was considered given the recent stent placement, but the persistent and progressive nature of troponin elevation, development of new wall motion abnormalities, and MRI findings of diffuse myocardial oedema extending beyond the stented territory made this less likely. Viral or other immune-mediated myocarditis unrelated to IgG4-RD could not be definitively excluded without endomyocardial biopsy; however, the patient’s prior diagnosis of probable IgG4-RD, temporal relationship to corticosteroid cessation, and clinical response to immunosuppression supported IgG4-RD as the most likely underlying aetiology.

The diagnostic challenge in this case was compounded by the limitations of relying solely on serology. Serum IgG4 concentrations may be normal during active disease, particularly during relapses or in organ-limited involvement, as well as with steroid therapy.^9^ Approximately 30% of patients with histopathologically confirmed IgG4-RD have normal serum IgG4 concentrations.^6,7^ Furthermore, elevated serum IgG4 can be seen in various non-IgG4-RD conditions including malignancies, other autoimmune diseases, and infection. In our patient, repeated angiography showing patent stents and preserved coronary anatomy prompted re-evaluation of the working diagnosis. Cardiac MRI provided critical evidence of myocardial inflammation with a non-ischaemic fibrosis pattern, supporting the clinical suspicion of IgG4-RD myocarditis and guiding immunosuppressive therapy initiation.

Treatment of IgG4-RD is not standardized; however, the mainstay of treatment is with corticosteroids followed by steroid-sparing immunosuppressive agents for maintenance therapy.^10,11^ For myocarditis associated with systemic autoimmune disorders, corticosteroids are generally used as first-line therapy.^8^ In this case, early re-initiation of corticosteroids followed by MMF maintenance was chosen based on the patient’s prior response to corticosteroids and need for long-term immunosuppression given the relapsing nature of her disease. The timing of immunosuppression initiation was guided by exclusion of active infection and the progressive nature of cardiac dysfunction despite optimal medical management. While the clinical and radiographic improvement following immunosuppression supports an inflammatory aetiology, it is important to acknowledge that spontaneous recovery of left ventricular function can occur in acute myocarditis cases, and stress-induced cardiomyopathy also typically resolves over weeks to months.^9,12,13^

Finally, this case reinforces the ACR/EULAR classification principle that exclusion criteria should serve as a guide to ensure alternative diagnoses are considered, rather than as a rigid checklist.^5^ Maintaining this flexibility is crucial when evaluating atypical cardiac presentations, particularly in patients with known systemic inflammatory diseases, to avoid missed or delayed diagnosis. The absence of tissue confirmation in this case limits diagnostic certainty, and the diagnosis should be considered probable or clinically inferred rather than definitive.

## Data Availability

No new data were generated or analysed in support of this research.

## Lead author biography

Emily Ellis is an internal medicine resident at Riverside Methodist Hospital in Columbus, OH. She graduated from Ohio University Heritage College of Medicine in Cleveland, OH.

## Notes

**Consent:** Written informed consent was obtained from the patient for publication of this case report and any accompanying images. A copy of the written consent is available for review by the Editor-in-Chief of this journal on request.

## Patient’s perspective

Patient’s perspective was not obtained for this case report.
